# EEG microstates are associated with music training experience

**DOI:** 10.3389/fnhum.2024.1434110

**Published:** 2024-07-25

**Authors:** Yihe Jiang, Maoping Zheng

**Affiliations:** ^1^Key Laboratory of Cognition and Personality (Ministry of Education), Southwest University, Chongqing, China; ^2^School of Psychology, Southwest University, Chongqing, China; ^3^School of Music, Southwest University, Chongqing, China

**Keywords:** musical training, EEG, microstates, resting-state, spatial clustering

## Abstract

**Background:**

Music training facilitates the development of individual cognitive functions and influences brain plasticity. A comprehensive understanding of the pathways and processes through which music affects the human brain, as well as the neurobiological mechanisms underlying human brain perception of music, is necessary to fully harness the plasticity that music offers for brain development.

**Aims:**

To investigate the resting-state electroencephalogram (EEG) activity of individuals with and without music training experience, and explore the microstate patterns of EEG signals.

**Method:**

In this study, an analysis of electroencephalogram (EEG) microstates from 57 participants yielded temporal parameters(mean duration, time coverage, occurrence, and transition probability)of four classic microstate categories (Categories A, B, C, and D) for two groups: those with music training experience and those without. Statistical analysis was conducted on these parameters between groups.

**Results:**

The results indicate that compared to individuals without music training experience, participants with music training experience exhibit significantly longer mean durations of microstate A, which is associated with speech processing. Additionally, they show a greater time coverage of microstate B, which is associated with visual processing. Transition probabilities from microstate A to microstate B were greater in participants with music training experience compared to those without. Conversely, transition probabilities from microstate A to microstate C and from microstate C to microstate D were greater in participants without music training experience.

**Conclusion:**

Our study found differences in characteristic parameters of certain microstates between individuals with and without music training experience. This suggests distinct brain activity patterns during tasks related to speech, vision, and attention regulation among individuals with varying levels of music training experience. These findings support an association between music training experience and specific neural activities. Furthermore, they endorse the hypothesis of music training experience influencing brain activity during resting states. Additionally, they imply a facilitative role of music training in tasks related to speech, vision, and attention regulation, providing initial evidence for further empirical investigation into the cognitive processes influenced by music training.

## 1 Introduction

Music training has been shown to significantly enhance brain structural and functional plasticity, thereby improving cognitive functions including memory, attention, and executive functions (Cheng et al., 2017), Groussard et al. (2014) conducted a study involving 44 non-musicians and amateur musicians, revealing that music training leads to gradual increases in gray matter volume in specific brain regions. These changes predominantly occur in the left hippocampus, right medial frontal and superior frontal regions, followed by the right insula and supplementary motor area, as well as the left superior temporal and posterior cingulate cortex. This suggests that music training can induce dynamic structural changes in the brain. Olszewska et al. (2021) reviewed the effects of music training on brain reorganization and neurobiological markers, highlighting functional and structural changes in the auditory and motor systems, emphasizing the emergence of a “musical brain” as a product of natural human neural diversity and training practices. Peretz and Zatorre (2005) further summarized the organizational structure of music processing in the brain, discussing core musical abilities shared by musicians and non-musicians, and separately examining the impact of music training on brain plasticity. They noted profound effects on brain structure and function despite cultural differences. These studies indicate that music training not only enhances musical skills but also profoundly influences the brain. Furthermore, research suggests Research indicates that regular music training not only enhances music-related skills such as music perception and performance abilities but also broadly strengthens non-musical cognitive domains (Steele et al., 2013; James et al., 2014; Moreno and Bidelman, 2014). For instance, long-term music training can enhance attention and processing speed (Roden et al., 2014), particularly showing prominent effects during the execution of complex tasks (Moreno et al., 2009). Furthermore, structural changes in the brain observed in adult participants through music training (Draganski and May, 2008) further underscore the importance of music training in promoting cognitive flexibility and higher cognitive functions. These studies emphasize the potential of music as an educational and therapeutic tool, providing robust scientific support for promoting overall cognitive health in individuals.

Investigating spontaneous brain activity provides a valuable approach for studying human cognition and behavior (Britz et al., 2010), in the past, traditional EEG and ERP signal processing has been mainly based on their time-domain and frequency-domain features (Maxwell et al., 2015; Wu et al., 2016). These methods often require defining regions of interest (ROI) encompassing several electrodes (Li et al., 2018). Therefore, these traditional methods of EEG signal analysis do not fully exploit the rich spatial information contained in EEG signals. EEG microstates refer to brief and relatively stable patterns of electrical potential distribution visible on an electroencephalogram (EEG), typically lasting from tens to hundreds of milliseconds. This approach considers multi-channel EEG recordings as a series of quasi-stable “microstates,” each characterized by a unique topography of potentials across the entire array of channels, can make up for the limitations of traditional EEG research methods. By simultaneously assessing signals from all regions of the cerebral cortex, this technique allows for the evaluation of large-scale brain network function, which is implicated in various neurobehavioral disorders associated with disruptions in these networks (Khanna et al., 2015). Electroencephalographic (EEG) microstates are considered transient stable phases of brain functional states directly linked to information processing and cognitive functions. This implies that by comparing microstates between musicians and non-musicians, fundamental changes in brain function due to music training can be directly observed.

Early research exploring the impact of musical training experience on brain functional microstates has begun to reveal differences in brain activity between musicians and non-musicians during auditory stimulus processing. Ott et al. (2011) using EEG microstate analysis, found that musicians exhibit more specialized patterns of brain activity when processing voiced and unvoiced auditory stimuli, suggesting that long-term musical training may optimize neural mechanisms for processing complex sounds. Building upon this, James et al. (2017) extended these findings with their electrical neuroimaging study, revealing associations between levels of musical expertise and mid-latency changes in brain activity, reflecting differences in higher cognitive functions during music processing among musicians. These studies provide a theoretical basis indicating that music training not only impacts brain structure but also finely tunes brain functional states during music information processing. They offer important insights for further investigating how music training refines brain electrophysiological activities. Moreover, recent studies have offered valuable insights into how musical training affects brain microstates. These studies suggest that musical training can alter brain structure and function and enhance the processing of music-related information. For instance, research by Bolduc et al. (2012) indirectly indicates that prolonged musical training might be associated with increased duration and stability of brain microstates by studying the relationship between brain capacity and functional outcomes in children with cerebellar malformations. Additionally, Fujioka (2006) provide direct evidence of how musical training influences brain development by observing changes in the auditory cortex responses of young children after a year of musical training. Further, research by Schön et al. (2004) reveals the link between musical training and language abilities, particularly how musical training enhances speech recognition and processing. These studies suggest that musical training may improve language processing abilities by affecting specific brain microstates. Together, these studies underscore the potential role of musical training in enhancing brain function and cognitive abilities. By combining behavioral tests and brain imaging techniques such as EEG and fMRI, researchers have gained a deeper understanding of how musical training intricately alters brain electrophysiological activity and the dynamics of microstates. These findings not only enhance our understanding of how music affects the brain but could also guide the development of future music-based therapeutic and educational interventions. While these studies have focused on differences in brain functional patterns between musicians and non-musicians, revealing that music training can alter the brain’s electrophysiological characteristics, such changes may be evidenced through analysis of EEG microstates. However, research specifically on how music training affects EEG microstates remains a relatively new area, requiring more experiments and data to gain a deeper understanding of how music training influences brain function and structure at the neural level. These findings hold significant implications for fields such as education, neuroscience, and psychology.

The brain’s momentary, global functional states are reflected through its electric field structure. Typically, a clustering analysis method is employed to extract four kinds of scalp surface electroencephalogram (EEG) structures, which can explain the temporal variations in their spontaneous EEG recordings. These four structures are referred to as EEG microstates A, B, C, and D classes (Milz et al., 2016; Tarailis et al., 2024; Zhang and Lyu, 2024). The source of microstate A is located on the left side of the occipital gyrus, insula, temporal lobe, and medial prefrontal cortex (mPFC) (Custo et al., 2017), primarily associated with changes in bilateral temporal parietal lobe cortex and middle temporal lobe cortex BOLD activation (Michel and Koenig, 2018). Microstate A is related to the level of brain arousal and speech auditory processing (Tarailis et al., 2024; Zhang and Lyu, 2024). Microstate B is significantly associated with BOLD changes in the striate and reticular cortex, as well as with bilateral occipital cortex BOLD deactivation closely related to the visual system (Michel and Koenig, 2018). Microstate B is related to psychological visualization of context (Bréchet et al., 2019) and visual processing associated with self-visualization and self-memory (Antonova et al., 2022; Tarailis et al., 2024). In a study involving repetitive transcranial magnetic stimulation of brain regions within the Default Mode Network (DMN) and Dorsal Attention Network, researchers found significant changes in the topography of microstate C following stimulation of the angular gyrus and intraparietal sulcus compared to pre-stimulation conditions. These changes were not observed after sham stimulation or stimulation of the temporoparietal junction. The researchers interpreted this as causal evidence linking the Dorsal Attention Network and DMN, indirectly mediated by a salience network (Croce et al., 2018). Therefore, microstate C was associated with self-related thoughts, mind-wandering, as well as emotional and interoceptive processing (Tarailis et al., 2024). Microstate D is associated with negative BOLD activation in the right ventral and dorsal areas of the frontal cortex and parietal cortex, closely related to attention networks (Britz et al., 2010). Microstate D is widely connected to default mode and dorsal attention networks (Spreckelmeyer et al., 2013), reflecting attention and cognitive control functions (Antonova et al., 2022; Tarailis et al., 2024), related to executive functions. The four classical microstate prototypes correspond to speech processing networks, visual networks, saliency default networks, and attention networks (Britz et al., 2010). The probability of transition between different categories of microstates can elucidate transitions within intrinsic brain functional networks (Khanna et al., 2015).

Therefore, this study aims to investigate the resting-state electroencephalogram (EEG) activity of individuals with and without music training experience and explore the microstate patterns of EEG signals. Additionally, since current research findings have not elucidated the differences in microstate temporal parameters between individuals with and without music training experience, this study seeks to deepen our understanding of how music training influences brain function and structure. Given that music training may have significant effects on cognition, perception, and motor control, comparing individuals with music training experience to those without it can further elucidate the potential benefits of music training on neural development. Moreover, we hypothesize that individuals with music training experience may exhibit longer microstates or show more significant differences in the occurrence frequency and time coverage of specific microstates. Furthermore, there may be correlations between music training experience and microstate characteristics, revealing potential mechanisms through which music training influences the dynamics of brain microstates.

## 2 Materials and methods

### 2.1 Participants

To achieve sufficient statistical power, we referenced previous studies (Bréchet et al., 2019; Korn et al., 2021; Antonova et al., 2022; Zhang et al., 2023) and also used G*Power 3.1.9.2 software beforehand to calculate the required sample size. The calculations for statistical power and effect size both yielded 0.8. The computed results indicate that 26 participants are needed per group. Therefore, we selected 57 college student participants based on previous related research (48 females, *M* = 20.68 ± 1.93 years old) were recruited through campus advertisements and online announcements. They were divided into two groups: Individuals with music training experience (27 participants) were undergraduate students majoring in music education at a university. They were concurrently studying vocal and instrumental (90% studied piano) music and had received over 5 years of music training and individuals without music training experience (30 participants, no formal music training). All participants were right-handed, had no history of psychiatric disorders, and had normal hearing and vision (corrected). Before the experiment, all participants completed a demographic questionnaire, signed informed consent forms, and received compensation upon completion of the experiment. This study was approved by the Ethics Committee of Southwest University (Licence No. H23116).

### 2.2 Procedure

The participants completed the experiment in a temperature-controlled and softly lit laboratory. They sat approximately 40–60 cm away from the screen. Resting-state EEG data collection lasted for a total of 10 min and 30 s. During the first 5 min and 30 s, participants focused on a fixation point (“+”). Following this, there was an additional 5-min period during which participants were instructed to keep their eyes closed, relax, remain awake, and avoid engaging in any specific thoughts. Previous research has indicated that closed-eye EEG data can provide more reliable spectral measurements of resting-state brain activity (Barry et al., 2007). Therefore, we analyzed the EEG data collected during the last 5 min when participants had their eyes closed.

### 2.3 EEG recording and preprocessing

The EEG signal was recorded using a 32-electrode elastic cap (Neuroscience Inc.), which was positioned according to the extended international 10–20 system. The specific electrode placements were at the following locations: Fp1, Fp2, F3, F4, C3, C4, P3, P4, O1, O2, F7, F8, T7, T8, P7, P8, Fz, Cz, Pz, Oz, FC1, FC2, CP1, CP2, FC5, FC6, CP5, CP6, AFz, CPz, POz, and Oz. The sampling rate was 1,024 Hz, and the impedance of all electrode sites was maintained below 10 kΩ. A reference electrode (REF) was used as the online reference, and bilateral mastoids served as the reference electrodes for offline analysis. Data processing was conducted using MATLAB R2019b and EEGLAB toolbox v2023.0.

Preprocessing of the data was performed as follows: channel positioning, segmentation (removal of non-useful periods), deletion of non-useful electrodes, bandpass filtering (2 ~ 20 Hz), notch filtering(48 ~ 52 Hz), and down the sampling rate from 1,024 Hz to 500 Hz. Then, manual data inspection for poor electrode interpolation, segmenting into 2-s epochs, removing segments with severe drift, applying Independent Component Analysis(ICA) and removing eye movement and head movement artifacts, removing extreme values(amplitudes exceeding ±100 μV), and adopting a whole-brain average reference.

### 2.4 EEG microstate analysis

Microstate analysis was conducted using Cartool software. Initially, EEG data were extracted from all available channels at the point of maximum Global Field Power (GFP) to ensure a better signal-to-noise ratio, focusing solely on data coinciding with GFP peaks. Subsequently, each participant’s data underwent analysis employing the Topographic Atomize & Agglomerate Hierarchical Clustering (T-AAHC) algorithm to ascertain the optimal number of categories and the corresponding templates for each dataset. Following this, the group average template topography was computed based on the optimal classification derived from each participant’s dataset. A predetermined number of 4 clusters was selected to facilitate comparability with prior studies. Finally, pertinent resting-state indicators were derived by amalgamating each participant’s EEG data with the group average template topographic map.

This study selected the average duration of each microstate (mean duration), the frequency of occurrence (occurrence, the average number of occurrences of each microstate category per second), time coverage (the total duration of each microstate category as a percentage of the total resting EEG), and the transition probability (the probability of transition from one microstate category to another)as characteristic parameter.

### 2.5 Statistics analysis

The regression analysis investigated how music training experience influences various characteristic parameters of microstates, including mean duration, time coverage, occurrence, and transition probabilities. Participants were classified into two groups based on their music training experience: those with music training experience and those without. Data collection involved recording the microstate feature parameters for each participant, which were then subjected to descriptive statistical analysis to determine the mean and standard deviation within each group. Assumption testing was conducted to ensure the data met the necessary criteria for parametric testing. Following this, independent samples t-tests were performed using IBM SPSS 27 software to compare the differences in microstate feature parameters between the two groups. The interpretation of these results focused on understanding the impact of music training on the brain’s microstate characteristics. Any significant differences found were discussed to elucidate how music training might influence brain dynamics, thereby offering insights into the relationship between music training experience and specific microstate features. This detailed approach ensured a thorough examination of the potential influence of music training on microstate parameters.

## 3 Results

**Figure 1:**
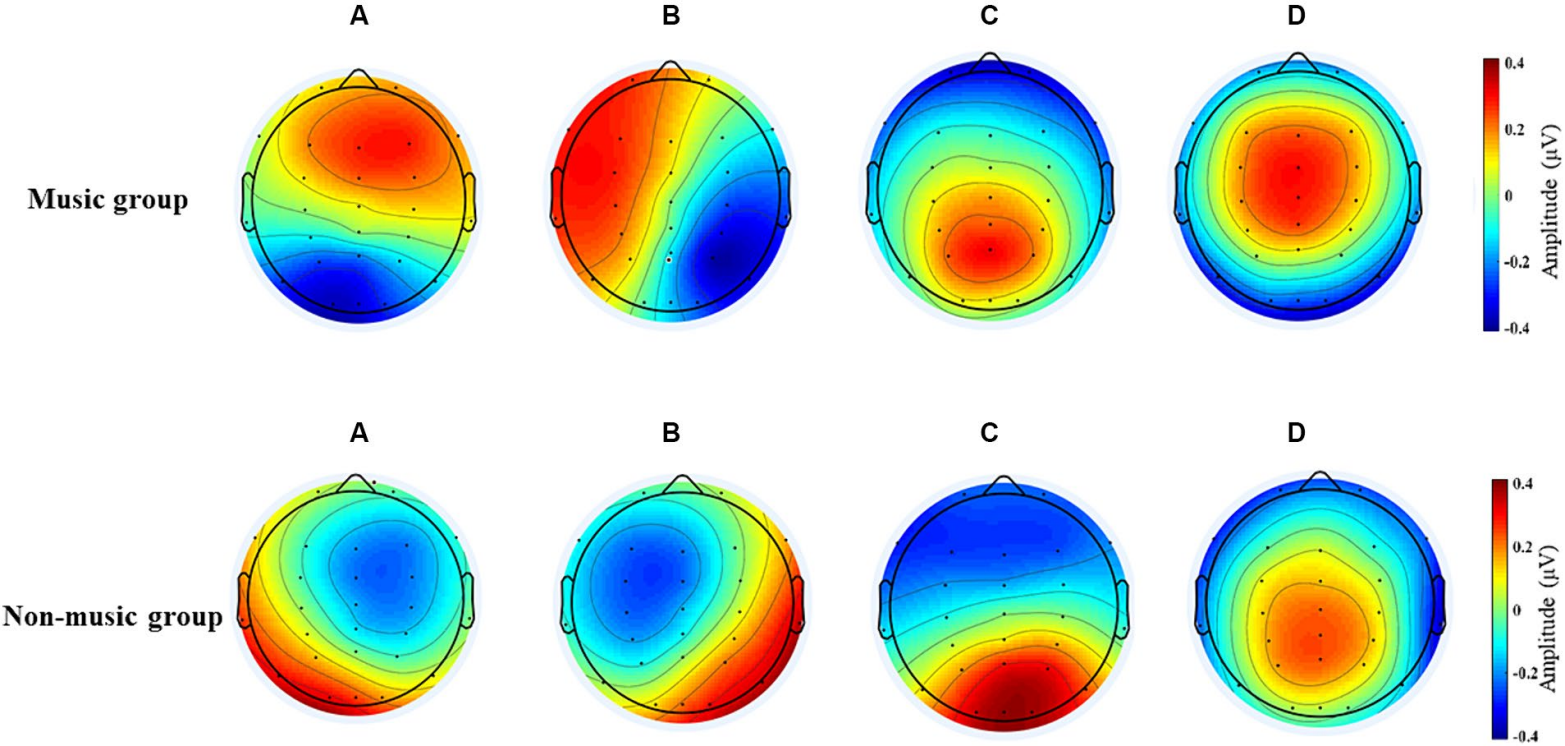
The four microstates recognized by k-means cluster analysis across subjects in the music and non-music groups.

Figure 1 displays four microstates were recognized by k-means cluster analysis across subjects in the group with music training experience and the group without music training experience. Based on the commonly used calculation method of microstate analysis in current research, we used the Global explained variance(GEV) to describe the variance proportion when explaining all EEG data based on the template map (Zhang et al., 2023; Zhou et al., 2023; Tarailis et al., 2024; Wu et al., 2024; Zhang and Lyu, 2024). In this study, the GEV for the four microstates was 67.3%.

Through regression analysis, we investigated the relationship between participants’ music training experience and the characteristics of EEG microstates. The results indicated that music training experience was significantly positively associated with the mean duration of microstate A(β = 0.289, R^2^ = 0.084, *p* = 0.029), the time coverage of microstate B(β = 0.377, R^2^ = 0.142, *p* = 0.004), the occurrence frequency of microstate B(β = 0.381, R^2^ = 0.145, *p* = 0.003), the occurrence of microstate D(β = 0.312, R^2^ = 0.097, *p* = 0.018), and the transition probabilities from microstate A to microstate B(β = 0.358, R^2^ = 0.128, *p* = 0.006). No music training experience was significantly negatively associated with the transition probabilities from microstate A to microstate C(β = −0.319, R^2^ = 0.102, *p* = 0.015) and the transition rate from microstate C to microstate D(β = −0.307, R^2^ = 0.094, *p* = 0.020).

**Figure 2:**
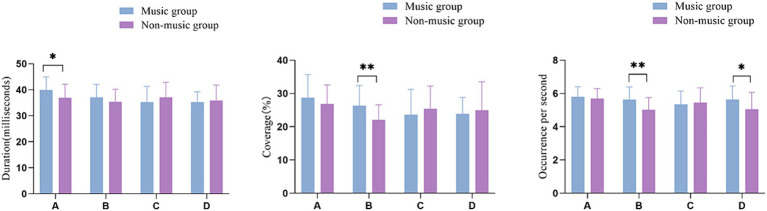
EEG microstate characteristics in groups with different music training experience. *p <0.05, **p <0.01.

By using the method of t-test, the microstate duration, coverage, occurrence per second, and microstate transition probabilities between the group with music training experience and the group without music training experience. Table 1 presents the mean values of four microstates in participants with and without music training experience. The analysis revealed that participants with music training experience had a longer duration of microstate A compared to those without music training experience (*p* = 0.029, *t* = −2.240, Cohen’s *d* = − 0.594). Additionally, participants with music training experience exhibited a greater time coverage of microstate B compared to those without music training experience (*p* = 0.004, *t* = −3.021, Cohen’s *d* = −0.801). Moreover, individuals with music training experience showed a higher occurrence per second of microstate B (*p* = 0.003, *t* = −3.058, Cohen’s *d* = −0.811) and D (*p* = 0.018, *t* = −2.435, Cohen’s *d* = −0.646) compared to those without music training experience. There was no significant difference in the change of microstate C between the two groups (Figure 2).

**Table 1:** Mean values of four microstates in participants with and without music training experience (*M ± SD*).

Microstate parameters	Musicians				Non-musicians			
	A	B	C	D	A	B	C	D
Duration	39.96 ± 4.99	37.12 ± 4.92	35.28 ± 6.01	35.31 ± 3.90	39.93 ± 5.20	35.40 ± 4.76	37.14 ± 5.74	35.86 ± 5.95
Occurrence	5.80 ± 0.60	5.62 ± 0.76	5.34 ± 0.80	5.64 ± 0.79	5.69 ± 0.59	5.01 ± 0.73	5.45 ± 0.88	5.05 ± 1.01
Coverage	28.76 ± 6.93	26.33 ± 6.08	23.61 ± 7.63	23.87 ± 4.92	26.89 ± 5.68	22.08 ± 4.51	25.41 ± 6.82	24.94 ± 8.57

**Figure 3:**
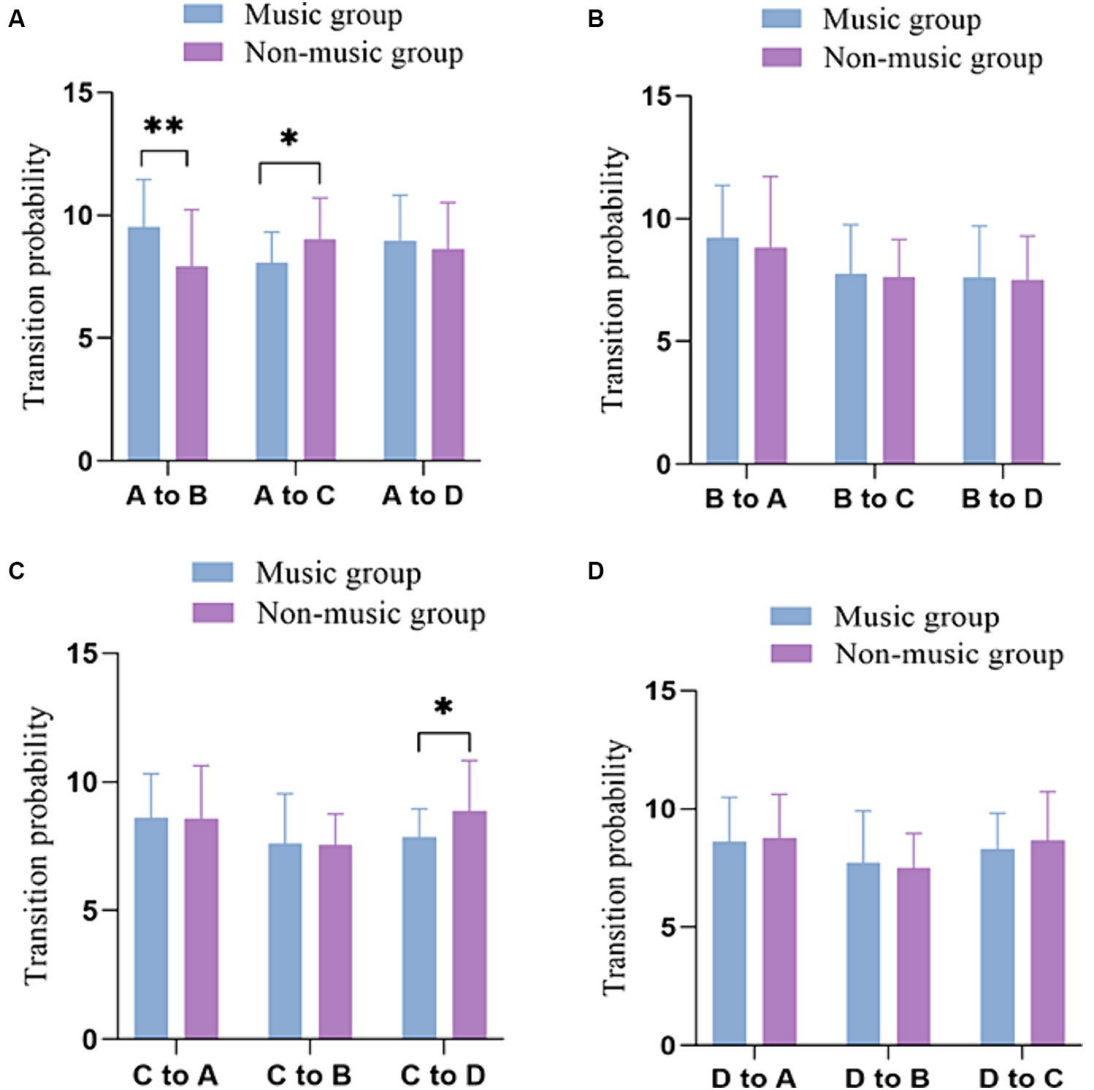
Comparison of transition probabilities of the four microstates in the music and non-music group. **(A)** The transition probabilities from microstate A to other microstates. **(B)** The transition probabilities from microstate B to other microstates. **(C)** The transition probabilities from microstate C to other microstates. **(D)** The transition probabilities from microstate D to other microstates. *p <0.05, **p <0.01.

In comparison, a t-test was conducted on the bidirectional transition probabilities of two groups of participants. Table 2 displays the Probability of transition between microstates in groups with and without music training experience. The results revealed that the music group participants exhibited a higher transition probability of conversion from microstate A to microstate B compared to the non-music group (*p* = 0.006, *t* = −2.841, Cohen’s *d* = −0.754). Conversely, the non-music group participants exhibited higher transition probability from microstate A to microstate C(*p* = 0.015, *t* = 2.500, Cohen’s *d* = 0.663) and from microstate C to microstate D(*p* = 0.020, *t* = 2.393, Cohen’s *d* = 0.635) compared to the music group participants (Figure 3).

**Table 2:** Probability of transition between microstates in groups with and without music training experience (%).

Microstate parameters	Musicians				Non-musicians			
	A	B	C	D	A	B	C	D
A	NA	9.534	8.071	8.971	NA	7.930	9.049	8.631
B	9.236	NA	7.747	7.610	8.838	NA	7.624	7.487
C	8.610	7.588	NA	8.877	8.575	7.558	NA	8.877
D	8.624	7.733	8.304	NA	8.764	7.492	8.668	NA

## 4 Discussion

This study investigated the influence of music training experience on the brain’s temporal network. The results indicate a significant positive correlation between music training experience and the mean duration of microstate A, the coverage ratio of microstate B, the frequency of occurrence of microstate D, and the transition probabilities from microstate A to microstate B. Conversely, there is a significant negative correlation between music training experience and the transition rate from microstate A to microstate C, as well as the transition rate from microstate C to microstate D. These findings suggest that individuals with music training experience exhibit enhanced temporal stability and efficiency in their brain network dynamics, particularly in the specific transitions and durations of distinct microstates, which may underlie improved cognitive and perceptual abilities associated with musical expertise (Zatorre et al., 2007; Herholz and Zatorre, 2012).

In previous studies, musicians have shown increased network volumes in brain regions associated with higher-order cognitive functions such as attention, memory, and executive functions, which are developed through music learning and performance. Conversely, they tend to exhibit decreased network volumes in regions linked to sensorimotor functions, likely due to the auditory and manual training emphasis in music education. This leads to structural changes in brain regions associated with these functions (James et al., 2014). In our study, participants with music training experience demonstrated longer average durations during microstate A, suggesting enhanced speech processing abilities, possibly indicating stronger attention or more efficient information processing among musicians (Wang et al., 2015). However, research has shown that microstate B is associated with visual networks (Seitzman et al., 2017), and there is a positive correlation between music training and spatial abilities (Wang et al., 2015). Using fMRI, Sluming et al. (2007) investigated cognitive performance in orchestral musicians and non-musicians during visual spatial tasks, revealing that musicians exhibited advanced abilities in three-dimensional mental rotation tasks, possibly due to well-developed neural circuits supporting such skills. Functional MRI also showed enhanced activation in the Broca’s area exclusively in orchestral musicians, crucial for sight-reading skills and motor sequence organization in musical performance, potentially supporting three-dimensional mental rotations. This suggests that sight-reading skill development in musicians alters neural circuit organization, offering broader cognitive benefits in non-musical visual spatial cognition. Moreover, studies indicate higher activation levels in the Broca’s area, right angular gyrus, and left inferior frontal gyrus among musicians compared to non-musicians. Individuals with music training also tend to perform better in tasks requiring spatial visualization abilities such as block design and mental rotation (Schellenberg, 2004; Pietsch and Jansen, 2012).

Participants with music training experience exhibit a higher time coverage in microstate B. This observation may imply the influence of music training experience on visual processing and visual network functionality, as well as its impact on the connections and regulatory mechanisms among different functional networks in the brain. The occurrence of microstates A and B may reflect the multimodal integration capabilities of individual brains (Oba et al., 2016; Zhang and Lyu, 2024), suggesting that participants with music training experience may be more effective at integrating sensory information. Additionally, this study also found that participants with music training experience exhibited a greater frequency of occurrence within unit time intervals in microstate D. Previous studies have confirmed that microstate D is associated with the dorsal attention network (DAN) (Britz et al., 2010), which governs external and attention-demanding cognitive functions (Corbetta and Shulman, 2002; Spreng et al., 2010). Therefore, this may suggest that music training helps to enhance the functionality of the individual’s dorsal attention network, enabling them to more effectively handle tasks requiring focused attention.

Participants with music training experience exhibited higher transition probabilities from microstate A to microstate B compared to those without music training experience, while transition rates from microstate A to microstate C and from microstate C to microstate D were lower in participants with music training experience than in those without. The higher transition probabilities from microstate A to microstate B in participants with music training experience may reflect changes in their focus of attention or allocation of attention. Music training may facilitate flexible responses of the brain to music and other stimuli, enabling individuals to transition more quickly from one cognitive state to another. Participants with music training experience exhibited lower transition rates from microstate A to microstate C compared to those without music training experience. This may indicate a more sensitive and automated attention allocation pattern towards external stimuli. Individuals without music training experience may be more inclined towards a more sensitive and automated attention allocation pattern towards external stimuli, whereas those with music training experience may be better able to maintain sustained attention to speech information while exhibiting fewer responses to salient stimuli in the environment (Roden et al., 2014). Moreover, participants with music training experience showed lower transition rates from microstate C to microstate D, suggesting that individuals without music training experience may more readily recruit the dorsal attention network to cope with the demands of external and attention-demanding cognitive tasks. In contrast, participants with music training experience may be better able to maintain activity in the saliency network, resulting in fewer transitions and more sustained attention to external stimuli. It is important to note that while microstate analysis provides valuable insights into brain electrical activity, its limitations should not be overlooked. Tarailis et al. (2024) pointed out that microstate analysis may not fully capture excitatory and inhibitory brain activity, which could potentially impact our study findings. Therefore, future research should integrate other analytical methods such as source localization techniques to gain a more comprehensive understanding of the neurophysiological characteristics represented by microstates.

These findings suggest that music training is associated with enhanced temporal stability and efficiency in brain network dynamics. Musicians spend more time in microstate A and B, and exhibit more frequent occurrences of microstate B and D, which align with literature indicating improved cognitive control and perceptual processing in musicians (Kraus and Chandrasekaran, 2010; Herholz and Zatorre, 2012; Smayda et al., 2015; Gustavson et al., 2021; Mittal et al., 2024). The increased duration within these microstates likely contributes to fewer transitions between microstates, reflecting a more stable and less fluctuating brain network at rest (Michel and Koenig, 2018). Furthermore, the differences in microstate transition dynamics may underlie the cognitive and perceptual benefits observed in musicians (Khanna et al., 2014; Strong and Midden, 2020). The increased stability in microstate A and coverage of microstate B may be linked to enhanced attentional and sensory processing capabilities (Lehmann et al., 2009; Gu et al., 2022; Kleinert et al., 2024), while the frequent occurrence of microstate D could be associated with more efficient auditory processing, crucial for musical training (Koelsch, 2011; D’Croz-Baron et al., 2021; Asha et al., 2024). Integrating these findings with existing literature provides a comprehensive understanding of how music training enhances brain network functionality. Future research should incorporate behavioral data to validate these interpretations further and explore the direct links between microstate dynamics and cognitive performance in musicians.

This study has several limitations. Firstly, different types of music training may influence changes in individual brain structure and function. Therefore, future research should differentiate the types of music training among participants. Secondly, the level of training may also impact changes in individual brain structure and function. Thus, in future studies, it will be important to categorize participants based on their training duration and longitudinally investigate the effects of music training on EEG activity. Finally, during the data collection process for resting-state EEG, we did not employ a method alternating between eyes-open and eyes-closed states. Although alternating between 5 min of eyes open and 5 min of eyes closed can minimize eye movement, it may lead to participant fatigue during the task. Future research should consider improvements in this aspect.

## 5 Conclusion

This study provides new evidence for the function and significance of EEG microstate identification, and for the first time, contrasts the microstate characteristics of individuals with and without music training experience. By examining the spontaneous resting-state EEG activity of individuals with and without music training experience and exploring the microstate patterns of EEG signals, our study found differences in characteristic parameters of certain microstates between individuals with and without music training experience. This may suggest the specificity of brain activity patterns during tasks related to speech, vision, and attention regulation among individuals with varying levels of music training experience. These findings support an association between music training experience and specific neural activities. These results provide preliminary evidence for further exploration into the effects of music training on brain activity, attention mechanisms, cognitive processing, individual differences, and brain plasticity. It is worth noting that the study offers a hypothetical explanation for the observed findings, specifically, we identified a relationship between microstate types and their characteristic parameters with music training experience. Zanesco et al. (2020) suggest that the function and significance of microstates during resting states are largely unknown, therefore, our study results require further validation in future experiments.

## Data availability statement

The raw data supporting the conclusions of this article will be made available by the authors, without undue reservation.

## Ethics statement

The study was approved by the Ethics Committee of Southwest University (Licence No. H23116). The studies were conducted in accordance with the local legislation and institutional requirements. The participants provided their written informed consent to participate in this study.

## Author contributions

YJ: Conceptualization, Data curation, Formal analysis, Investigation, Methodology, Project administration, Resources, Software, Supervision, Validation, Visualization, Writing – original draft, Writing – review & editing. MZ: Funding acquisition, Project administration, Resources, Supervision, Writing – review & editing.
